# Strengthening Long-Term Care Workplace Culture and Worker Voice Through the Coach Approach Intervention

**DOI:** 10.1093/geroni/igaf122.008

**Published:** 2025-12-31

**Authors:** Kezia Scales, Emily Dieppa Colo, McKayla Brady

**Affiliations:** PHI, New York, New York, United States; Paraprofessional Healthcare Institute, New York, New York, United States; Paraprofessional Healthcare Institute, New York, New York, United States

## Abstract

Communication challenges in long-term care organizations can strain relationships, curtail workers’ engagement and contributions, and impact care delivery and outcomes. PHI, a national organization focused on the direct care workforce in long-term care, developed the Coach Approach organizational change model to enhance communication skills among all members of the care team in long-term care settings toward a goal of building strong, respectful relationships that foster collaboration and collective problem-solving. The Coach Approach model comprises three components: executive coaching, supervisory training (“Coaching Supervision”), and frontline worker training (“Coaching Communication”). For this evaluation study, PHI contracted with a large national home care provider to adapt and deliver the Coach Approach intervention in a hybrid format to their full staff between January and August 2024. Findings from the evaluation revealed high levels of satisfaction with the training, and 100% of supervisors and 97% of frontline staff reported that they have used the skills they acquired. Findings also indicated that the Coach Approach helps care team members appreciate one another’s perspectives and work together more effectively. The evaluation results demonstrate the value of implementing communication training across all staffing levels within a large, multi-site long-term care organization with implications for improving job quality and care quality for older adults and people with disabilities being served.

